# Tryptophanase-Catalyzed l-Tryptophan Synthesis from d-Serine in the Presence of Diammonium Hydrogen Phosphate

**DOI:** 10.3390/ijms10062578

**Published:** 2009-06-03

**Authors:** Akihiko Shimada, Haruka Ozaki, Takeshi Saito, Fujii Noriko

**Affiliations:** 1 Sustainable Environmental Studies, Graduate School of Life and Environmental Sciences, University of Tsukuba, Tsukuba, Ibaraki 305-8572, Japan; 2 Research Reactor Institute, Kyoto University, Kumatori, Osaka 590-0494, Japan

**Keywords:** tryptophanase, diammonium hydrogen phosphate, d-serine, tryptophan synthesis, stereospecific change

## Abstract

Tryptophanase, an enzyme with extreme absolute stereospecificity for optically active stereoisomers, catalyzes the synthesis of l-tryptophan from l-serine and indole through a β-substitution mechanism of the ping-pong type, and has no activity on d-serine. We previously reported that tryptophanase changed its stereospecificity to degrade d-tryptophan in highly concentrated diammonium hydrogen phosphate, (NH_4_)_2_HPO_4_ solution. The present study provided the same stereospecific change seen in the d-tryptophan degradation reaction also occurs in tryptophan synthesis from d-serine. Tryptophanase became active to d-serine to synthesize l-tryptophan in the presence of diammonium hydrogen phosphate. This reaction has never been reported before. d-serine seems to undergo β-replacement *via* an enzyme-bonded α-aminoacylate intermediate to yield l-tryptophan.

## 1. Introduction

Life is regarded as a highly-organized precision structure made up of homochiral biomolecules. For example, if a polypeptide is mixed with l- and d- amino acids, it is no longer possible for it to build an ultraprecise structure because the racemic mixture of optical antipodes cannot elongate the polypeptide chain [1]. Amino acids, which can be regarded as component parts for all polypeptides, must be homogeneous to assemble a regularly-structured polypeptide. Life might have not existed if the components had been enantiometrically heterogeneous in an earlier stage of birth of life. Life’s first strategy to build up highly organized biomacromolecules is to homogenize amino acids or sugars as the components. However, even if asymmetric amino acids and sugars formed on earth before life had produced only slight enantiomeric excess, any slight excess would have been easily degraded by racemization in water [2]. Perhaps some elaborate contrivance is required to produce homochiral amino acid molecules enough to synthesize the polypeptide. At least it should have a systematic mechanism with automatically developing chiral homogeneity [3], and additionally should be a very sophisticated mechanism that selects and delivers the right enantiomeric type to the right place together with the exclusion of the opposite enantiomer to synthesize regularly-structured macromolecules. By the time catalytically active early polypeptides, i.e., primitive enzymes, were present, the switch to the exclusive use of l-amino acids might already have been made. If early polypeptides were thus synthesized by spontaneous abiotic processes in a primitive racemic environment, the mechanism of homochirality may have already been incorporated into them, and their descendants may be traced to present enzymes. Such mechanism is involved in enzyme stereospecificity in extant life. It must be absolutely stable because unstable stereospecificity would mean the death of life. We can thus say life does not exist without homochirality, i.e. no homochirality – no life [4]. This principle is so fundamental that it is inseparably associated with the origins of life. Chiral homogeneity, which is directly linked to the enzyme stereospecificity, has intrigued scientists for many years, though there has been no general consensus as yet to the origins of homochirality. More detailed studies on the enzyme stereospecificity should be made to elucidate the origin of homochirality.

We have so far studied about stereospecificity of tryptophanase, with the aim of obtaining any clue to answer that problem. Tryptophanase is an enzyme with extremely tight stereospecificity, cleaving l-tryptophan into indole, pyruvate and ammonia, and displaying no activity on d-tryptophan under usual conditions. However, it becomes active toward d-tryptophan degradation in highly concentrated diammonium hydrogen phosphate solutions. This reaction was discovered by sheer accident when we researched tryptophanase stereoselectivity. The details of this reaction are presented elsewhere [5–8]. These results obtained from d-tryptophan degradation reaction indicated the enzyme stereospecificity was flexible. However, in the studies focused on the origins of life, synthesis reaction has greater significance over degradation reaction because life is born by the accumulation of countless numbers of synthetic reactions. For the purpose of the present study, it is more valuable to study the flexibility of the stereospecificity in the synthesis reaction. Tryptophanase can synthesize l-tryptophan from l-serine and indole according to the reaction equation:

$$\text{L}-\text{serine}\;+\;\text{indole}\;\to \;\text{L}-\text{tryptophan}\;+\;{\text{H}}_{2}\text{O}$$

Tryptophanse has absolute stereoselectivity in this synthesis reaction and therefore no activity on d-serine at all. The β-substitution mechanism of l-tryptophan synthesis from indole and l-serine is the ping-pong type mechanism [9]. Morino and Snell reported an enzyme-bounded α-aminoacylate intermediate was important in tryptophan synthesis from L-serine [10]. Although diammonium hydrogen phosphate gives small reversible conformational change to induce the d-tryptophan degradation reaction, it appears reasonable to deduce that tryptophanase receives the same stereospecific change from diammonium hydrogen phosphate in tryptophan synthesis reaction, too. Thus our studies have led us to consider the possibility of tryptophan synthesis from d-serine by flexible tryptophanase stereospecificity *via* the same mechanism used in the d-tryptophan degradation. This paper reports d-serine reacts with tryptophanase as a substrate for l-tryptophan synthesis in the presence of diammonium hydrogen phosphate.

## 2. Results and Discussion

### 2.1. Reactant product developed on cellulose thin layer chromatography

All proteinic amino acids can be resolved on cellulose thin layer chromatography at lower temperatures using development solvents such as *n*-butanol/pyridine/water or ethanol/pyridine/water [11,12]. Aromatic or heterocyclic amino acids with bulky side chains are generally resolved more easily than aliphatic amino acids. Resolution factor ΔR_f_′ of the former and the latter are 0.1 ~ 0.2 and 0.01 ~ 0.03, respectively, where ΔR_f_′ is given by the equation:

$$\Delta {\text{R}}_{\text{f}}^{\prime }=\Delta {\text{R}}_{\text{f}}/{\text{mR}}_{\text{f}}$$

where ΔR_f_ is the difference between the R_f_ value of d- and l-amino acids, and mR_f_ is the mean R_f_ value between d- and l- amino acids [13].

**Figure 1.**
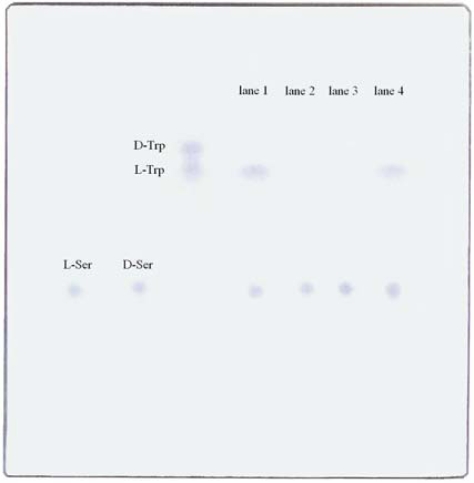
Thin layer chromatogram of the tryptophan synthesized from l- or d-serine by tryptophanase. For comparison, d-tryptophan, l-tryptophan, d-serine and l-serine were developed in the left half. Reaction products were developed in the right half. Lane1: l-serine + indole + tryptophanase in a potassium phosphate buffer; lane 2: d-serine + indole + 20 % saturation diammoniumhydrogen phosphate in a potassium phosphate buffer; lane 3: d-serine + indole + tryptophanase in a potassium phosphate buffer; lane 4: d-serine + indole + tryptophanase + 20 % saturation diammoniumhydrogen phosphate in a potassium phosphate buffer.

As seen in the left half of Figure 1, the ΔR_f_′ values of d, l-tryptophan and d,l-serine were calculated to be 0.12 and 0.03, respectively. Reactant products were analyzed in the right half lanes.

Two ninhydrin-colored purple spots, specific to amino acids, were seen in lane 1. R_f_ of the upper and lower spots was equal to l-tryptophan and l-serine, respectively. These results showed that l-tryptophan was synthesized from l-serine and indole by tryptophanase, as is well known. d-serine was used in lanes 2 – 4. Lane 2 reacted with indole + d-serine at 20 % saturation concentration of diammonium hydrogen phosphate in the absence of tryptophanase, and lane 3 reacted with indole + d-serine + tryptophanase in the absence of diammonium hydrogen phosphate. Both lanes did not present tryptophan. In lane 4, tryptophanase reacted with d-serine and indole, together with 20% saturation diammonium hydrogen phosphate. It was in lane 4 that tryptophan appeared. The enzyme could not synthesize tryptophan from d-serine without diammonium hydrogen phosphate.

### 2.2. Optimal concentration of diammoniumhydrogen phosphate

**Figure 2.**
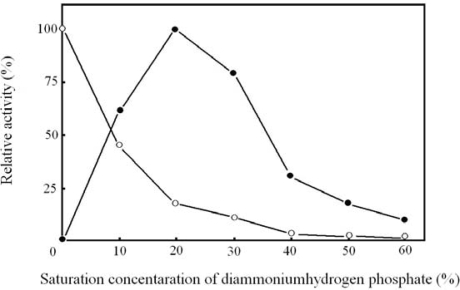
Tryptophan synthesis from l- or d-serine against diammonium hydrogen phosphate saturation concentration. ○: l-serine, •: d-serine.

Tryptophan synthesis from l- or d-serine was affected by diammonium hydrogen phosphate. After the products for each saturation concentration of diammonium hydrogen phosphate were resolved on a column to obtain a chromatogram, tryptophanase activity on tryptophan synthesis was calculated from a peak area at a retention time of l-tryptophan on that chromatogram. Any peak was not seen at or near a retention time of d-tryptophan in the absence or presence of diammonium hydrogen phosphate. Tryptophanase strictly upholds the basic principle of l-amino acid selection and d-amino acid exclusion in tryptophan synthesis. Figure 2 shows the relative activity that defines maximum activity on tryptophan synthesis from l- or d-serine as 100 %. When l-serine was substrate, the activity decreased with increasing diammonium hydrogen phosphate. The maximum activity was provided in the absence of diammonium hydrogen phosphate. Diammonium hydrogen phosphate served as an inhibitor of tryptophanase in this case. On the other hand, diammonium hydrogen phosphate served as an activator on tryptophan synthesis from d-serine. Activity on tryptophan synthesis increased with diammonium hydrogen phosphate, reaching the maximum activity at 20 % saturation concentration. Optimal concentration of diammonium hydrogen phosphate was used to analyze tryptophan synthesized from l- or d-serine.

### 2.3. Effects of other salts on tryptophan synthesis

Diammonium hydrogen phosphate was essential for tryptophan synthesis from d-serine. Since diammonium hydrogen phosphate consists of ammonium and phosphate ions, the effects their ions have on tryptophan synthesis were investigated using other salts, including either of the two ions in addition to NaCl and seawater, shown in Table 1. All salts were analyzed at a saturation concentration of 20%. The reaction product was developed on cellulose thin layer chromatography with development solvent, colored with ninhydrin and compared to the R_f_ value of tryptophan. Every salt tested had no activity on tryptophan synthesis except diammonium hydrogen phosphate [(NH_4_) _2_HPO_4_] as indicated in Table 1. It was only diammonium hydrogen phosphate that could produce tryptophan from d-serine. This activity was evoked not by ammonium or phosphate ions, but by some sort of biochemical property peculiar to diammonium hydrogen phosphate. As for the causes of this reaction, some have been considered. For example, we can hypothesize diammonium hydrogen phosphate can either give subtle conformational change to tryptophanase, or have a positive influence by promoting binding with d-serine and tryptophanase [14]. No matter what the cause may be, diammonium hydrogen phosphate is currently the only salt that makes it possible to synthesize tryptophan from d-serine.

### 2.4. Analyses of reaction product with resolution column by UV monitor

**Figure 3.**
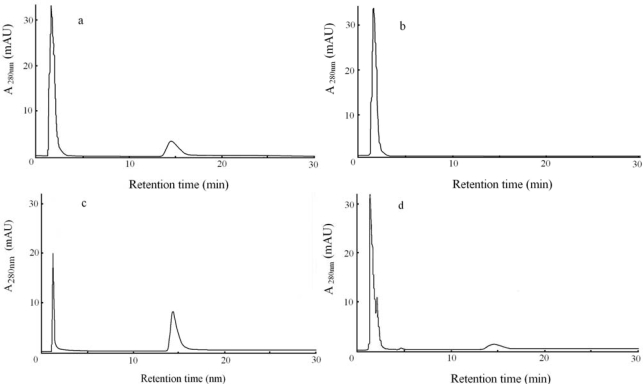
Resolution chromatograms of the reactant products (monitored by UV detection at λ = 280 nm). (a) Advanced resolution chromatography was carried out to determine a retention time of l-tryptophan. (b) There is no tryptophan peak in the absence of tryptophanase. (c) l-tryptophan (a peak at 14.6 min) was synthesized from l-serine and indole by tryptophanase in a potassium phosphate buffer solution. (d) Tryptophan was synthesized from d-serine and indole by tryptophanase in the presence of diammonium hydrogen phosphate.

All thin layer chromatography analyses showed that the reaction product was an amino acid with R_f_ equal to l-tryptophan. HPLC analyses by means of resolution column were needed for the product so that we could determine what kind of amino acid and optical isomeric form it is. The product was thus eluted on a resolution column to investigate its retention time. Reactions with l- or d-serine were performed under each optimal saturation concentration of diammonium hydrogen phosphate. Unwanted trash substances mixed with indole, diammonium hydrogen phosphate and pyridoxal 5′-phosphate were eliminated at the first sharp peak near void volume, seen in Figure 3a, and thereafter tryptophan was eluted. Both of l- and d-serine were hidden behind this large peak because of retention time around 2 min. A retention time of l-tryptophan was determined as 14.6 min (that of d-tryptophan was 11.4 min.). In Figure 3b, tryptophan was not produced at all because there was no tryptophanase, regardless if diammonium hydrogen phosphate, d-serine and indole were supplied. Figure 3c shows l-tryptophan synthesized from l-serine in potassium phosphate buffer solution. Tryptophanase activity on tryptophan synthesis from l-serine is maximal when diammonium hydrogen phosphate is absent. A smaller peak was detected at a retention time of 14.6 min in Figure 3d, seemingly corresponding to l-tryptophan on the basis of its retention time. This peak was further examined with a CD detector to confirm an optical isomeric type. Tryptophan was not synthesized until diammonium hydrogen phosphate was supplied in the presence of d-serine and indole. This peak area was 6% of one of l-tryptophan in Figure 3c. Tryptophanase activity on d-tryptophan degradation was reported to be 20% of that of l-tryptophan degradation in the previous study [14]. Tryptophanase activity on tryptophan synthesis from d-serine became less efficient than that on d-tryptophan degradation. This result indicates synthesis has more absolute stereospecificity than degradation.

### 2.5. Determination of optical isomeric form by circular dichroism

**Figure 4.**
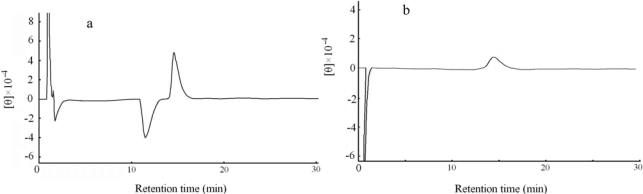
Detection of the reactant product with CD detector. (a) d, l-tryptophan as a standard substance was eluted onto a resolution column Crownpack CR (+). (b) The tryptophan synthesized was eluted onto the same column. Optical isomeric form of the tryptophan was established to be l type.

Optical isomeric form of the tryptophan synthesized was confirmed absolutely by measuring ellipticity with a CD detector. dl-tryptophan was resolved as a reference in Figure 4a. d-tryptophan appeared on the negative side in ellipticity at a retention time of 11.4 min, but l-tryptophan appeared on the positive side at 14.6 min. The reactant product synthesized from d-serine was resolved in Figure 4b; it was positioned at a retention time of 14.6 min on the positive side of the ellipticity, and this result also established that the reaction product was l-tryptophan.

### 2.6. Diammoniumhydrogen phosphate efficacy on tryptophan synthesis

We have so far studied tryptophanase, which is an enzyme with extremely tight stereospecificity, cleaving l-tryptophan into indole, pyruvate and ammonia by α, β-elimination, and having no effect on d-tryptophan under ordinary conditions. However, it becomes active to d-tryptophan in highly concentrated diammonium hydrogen phosphate solution. This result led us to doubts regarding the absolute exclusive selectivity of enzyme stereospecificity, as the results suggest that enzyme stereospecificity may be more flexible than we previously thought. Tryptophanase is inactive on d-tryptophan in the absence of diammonium hydrogen phosphate, so if you see only this, you may be led to conclude that it does not interact with d-tryptophan. In fact, quite the opposite was true. Previous kinetic studies indicated d-tryptophan competitively inhibited l-tryptophan degradation without diammonium hydrogen phosphate [15]. This indicates d-tryptophan binds to the active site of tryptophanse in the absence of diammonium hydrogen phosphate, but is not catalyzed. Circular dichroism and fluorescence spectrophotometric analyses indicated diammonium hydrogen phosphate brought about a mild change in tryptophanase stereostructure [16]. Subtle conformational change made it possible to catalyze d-tryptophan. Flexible stereospecificity is probably due to trivial conformational change. The barrier separating the two antipodes seems to be lower in tryptophan degradation reaction than we imagine. However, enzyme stereospecificity is considered more absolute in synthesis reaction than degradation reaction. The latter reaction, a catabolic process, purposes to obtain energy and is not concerned with the final metabolite. As a general trend, degradative enzyme is easier to accept D-amino acid than synthetic enzyme [17]. On the contrary, the former, an anabolic process, focuses on the final product because the metabolite produced is destined to be incorporated into the living body. Accordingly, the type of steric configuration or the chemical characteristics becomes critical. For example, enantiospecific influence on drug’s metabolism is not negligible in clinical level [18]. It is never allowed to incorporate the final product with the opposite enantiomeric type as living materials. Enzyme stereoselectivity should make a rigid distinction between enantiometrically d type and l type in a synthesis reaction more so than in a degradation reaction. Consequently, more absolute stereospecificity in the synthesis reaction makes it difficult to catalyze d-amino acid. Nevertheless we cannot still preclude the possibility on tryptophan synthesis from d-serine. Logically thinking, there is no problem at all if d-serine binds with the active site of tryptophanase in the same way as d-tryptophan. If so, there would be no surprise even if d-serine serves as a substrate for tryptophan synthesis. Thus we discuss in the next section on the possibility of binding with d-serine and tryptophanase based on the results obtained.

### 2.7. Speculation of l-tryptophan synthesis pathway from d-serine

Tryptophanase can synthesize l-tryptophan from l-serine and indole by β-substitution through the ping-pong type reaction mechanism. l-serine, after combining with tryptophanse, looses a hydroxyl group to yield an enzyme-bounded α-aminoacylate intermediate (E·CH_2_=C(NH_2_)COOH, E: tryptophanase) [10]. The intermediate can add indole to produce l-tryptophan by reversal against degradation [19,20]. In this synthesis reaction, there is no possibility that d-serine serves as substrate [21]. However, d-serine can be built into l-tryptophan in the presence of diammonium hydrogen phosphate. This shows d-serine binds to the active site of tryptophanase and forms an enzyme-substrate complex. In other paper, the activity of l-serine dehydratase in cleaving l-serine is competitively inhibited by d-serine, indicating that d-serine can occupy an active site on the enzyme [22]. It may well be that the same activity occurs in d-serine. In preliminary experiment, we found d-serine inhibited tryptophanase in a d-tryptophan-degraded reaction in the presence of diammonium hydrogen phosphate (data not provided here). Additionally, we have described above that d-tryptophan can bind with tryptophanase in the absence of diammonium hydrogen phosphate. These results suggest d-serine binds to the same binding site as d-tryptophan within the active site of tryptophanase, and also the existence of enzyme-substrate complex common to d-serine and d-tryptophan. We think it is the above E·CH_2_=C(NH_2_)COOH. It is an important intermediate for both l-tryptophan degradation and tryptophan synthesis from l-serine, too. In other words, the formation of the intermediate is the key of tryptophan synthesis from d-serine. Although the present study has remained unidentifed it, it is meaningful for further studies to speculate a reaction pathway of tryptophan synthesis from d-serine. Since tryptophanase cannot interact with d-serine in ordinary condition, it does not form E·CH_2_=C(NH_2_)COOH complex. When d-serine binds with tryptophanase in the presence of diammonium hydrogen phosphate, E·CH_2_=C(NH_2_)COOH complex is formed. Synthesis reaction was lower than degradation reaction on reaction efficiency. Perhaps the former is more resistant to this complex formation than the latter. The complex is followed by tryptophan formation in the presence of indole or by pyruvate formation in the absence of indole. The barrier discriminating between d- and l-amino acids, which serves as the driving force behind chiral homogeneity, is kept steady and strong in the active site. Diammonium hydrogen phosphate lowers the barrier through subtle conformational change of the active site. We think this study may present the key to solve the current disputes that surround the origin of homochirality.

## 3. Experimental Section

### 3.1. Saturated solutions of diammonium phosphate and other salts

Diammonium hydrogen phosphate [(NH_4_) _2_HPO_4_] was dissolved completely in 80 – 85 °C water and slowly cooled to room temperature. This concentrated salt solution was left at room temperature for about a week until crystals were deposited. All saturated solutions were used for experiments after crystallization finished completely. The saturated solutions thus prepared were defined as a 100 % saturation concentration. Each salt solution was diluted to the required saturation concentration with 100 mM potassium phosphate buffer (pH 8.3) prior to the experiment; e.g., diammoniumhydrogen phosphate solution with a 20 % saturation concentration was prepared by adding 4 parts potassium phosphate buffer to 1 part 100 % saturated diammonium hydrogen phosphate solution. Saturated solutions of other salts including ammonium or phosphates, i.e. NH_4_Cl, NH_4_NO_3_, ammonium dihydrogen phosphate (NH_4_H_2_PO_4_), (NH_4_)_2_CO_3_, K_2_HPO_4_, Na_2_HPO_4_, and NaCl, were prepared in the same way. Seawater was used as is.

### 3.2. Reaction mixture and reagents

l-Serine, d-serine, l-tryptophan and d-tryptophan were purchased from Peptide Institute Inc. (Osaka, Japan), indole from Wako Pure Chemical Industries (Osaka, Japan), pyridoxal 5′-phosphate from Nakalai Tesque Inc. (Kyoto, Japan), and apotryptophanase (tryptophanase), from Sigma-Aldrich Co. (St. Louis, MO, USA). The apotryptophanase was easily soluble in aqueous solution and SDS-PAGE shows a single band at MW 55,000, indicating that apotryptophanase was completely pure. 1 mg of apotryptophanase released 75 to 150 μg of indole from l-Trp at 10 min with pH 8.3 at 37 °C in the presence of 0.04 mM of pyridoxal 5′-phosphate. 100 mM potassium phosphate buffer of pH 8.3 was always used as standard buffer solution. Unless otherwise noted, all chemicals were high reagent grade, purchased from Wako Pure Chemical Industries. All glassware was washed by soaking for more than three days in a detergent, Clean 99 CL, and were thoroughly rinsed and then dried in an oven. All aqueous solutions were prepared from deionized and ceramics-distilled water.

Reaction mixtures included 0.2 mM of pyridoxal 5′-phosphate, 4.8 mM of l-serine or d-serine, and 2.7 mM of indole; 0.23 μM of tryptophanase. Saturation concentration of diammonium hydrogen phosphate was prepared from 0 to 60 %. 20% saturation concentration was used to analyze tryptophan synthesized from d-serine with HPLC. The effect of the different salts on tryptophanase activity toward d-serine was examined at 20% saturation concentration with each salt. A total volume of the reaction mixture was 2 mL per tube. Reactions were conducted at 37 °C for 6 hr in a Dry Thermo Unit DTU-1B (Taitec, Tokyo, Japan). The reaction was stopped by adding 2 mL of n-butanol for thin layer chromatographic analyses or by icing at 0 °C for high pressure liquid chromatographic analyses. The former was vigorously mixed, and then immediately centrifuged at approximately 1,000 g for 10 min. After centrifugation, the reaction mixture was separated into two phases. The supernatant was extracted, dried over several days at 60 °C in the oven to concentrate the tryptophan produced. The concentrated aliquot was applied for tryptophan analyses on cellulose thin layer chromatography.

### 3.3. Thin layer chromatographic analyses

The reactant product can be resolved with cellulose thin layer chromatography [23–25]. The composition of developing solvent is 1 part pyridine with 1 part *n*-butanol and 1 part water. All cellulose thin layer plates (Funacell SF 10 × 10 cm, Code No. FC-1010, Funakoshi Co., Tokyo, Japan) were triply predeveloped by washing with this same solvent to remove possible contaminants that can show unfavorable effects on development and the ninhydrin chromogenic reaction. The plates were washed and dried in the oven at 50 °C for 1 hr, stored in a plastic desiccator until use with silica gel. 5 μL of the aliquot that was soaked up by capillary action into a 10μℓ-ringcaps was spotted onto the cellulose this layer plates, which were ascendingly developed at a constant low temperature of 5 °C in a refrigerator. After development, the plates were dried up, and immediately sprayed by ninhydrin reagent for colorimetry. The R_f_ value of purple spot was compared to that of d- or l-tryptophan.

### 3.4. Tryptophan analyses by use of high pressure liquid chromatography (HPLC)

The reactant products were resolved on a Crownpack CR (+) Resolution HPLC column (Daicel Industry Ltd., Tokyo, Japan) to be analyzed with UV and CD monitor. Perchloric acid was added dropwise to a few liters of distilled water with vigorous stirring at room temperature until pH decreased to 2.0, and used as an eluting buffer for HPLC. The sample cooled at 0 °C with ice was filtered through Ultrafree-MC 5000 NMWL Filter Unit (Millipore Co. Bedford, MA, USA), immediately injected into a Crownpack CR (+) Resolution HPLC column, and resolved at a flow rate of 1.0 mL/min with the eluting buffer running with an HPLC pump (635 Liquid Chromatogram, Hitachi, Ltd., Tokyo, Japan) through a Degasser (DG-980-50, Jasco, Tokyo, Japan). The eluant was monitored with an UV monitor (1040A HPLC Detection System, Hewlett-Packard, CA, USA) for UV absorbance at a wavelength of λ = 280 nm. Absorbance was expressed in arbitrary unit (milliabsorbance unit; mAU). The retention time of d- and l-tryptophan was 11.4 and 14.6 min, respectively. Tryptophanase activity on tryptophan synthesis from l- or d-serine was calculated from a peak area of l-tryptophan on HPLC chromatogram, compared against saturation concentration of diammonium hydrogen phosphate. Indole, diammonium hydrogen phosphate and pyridoxal 5′-phosphate were eluted as a sharp large peak at or near void volume (retention time between 1 – 3 min). For this result, both d- and l-serine (retention time of 1.7 min for d-serine and 2.4 min for L-serine) were hidden behind the peak to be undetectable.

Circular dichroism (CD) is the most effective method for determining the enantiomeric form of tryptophan. As soon as tryptophan involved in the reactant products was resolved on the HPLC column, the optical isomeric form of tryptophan was identified with a CD detector (CD-1595 detector, Jasco, Tokyo, Japan). Ellipticity of the eluting buffer was set up to zero at a wavelength of λ = 233 nm, so that the ellipticity of d-Trp and l-Trp had a negative and positive peak, respectively. Vertical axis on chromatograms was represented in arbitrary unit of ellipticity [θ].

## 4. Conclusions

The only active enantiomeric form of tryptophan and serine is the l type in α-, β-eliminations and β-substitution reactions of tryptophanase under ordinary physiological saline concentrations because the enzyme has absolute stereospecificity. However, concentrated diammonium hydrogen phosphate can change tryptophanase stereoselectivity. When d-serine is added in a tryptophan synthesis reaction in the presence of diammonium hydrogen phosphate, it can serve as substrate to be synthesized into l-tryptophan. Although absolute stereoselectivity is generally displayed in a synthesis reaction more so than in a degradation reaction, the present result also reconfirms that synthesis has less efficient activity than degradation. In addition we speculate l-tryptophan is synthesized from d-serine by tryptophanase *via* enzyme-bounded α-aminoacylate intermediate, which plays a key role in l-tryptophan synthesis from l-serine. It appears reasonable to think the tryptophanase stereoselectivity is more flexible than we always image. Perhaps the exclusive stereoselective mechanism of l-amino acid from d-amino acid will be driven by the slight difference in stereostructure. The mechanism of changing stereospecificity on tryptophanase has still remained ambiguous, but we are confident that it will offer valuable information and clue to the origin of homochirality.

## Figures and Tables

**Table 1.** Effects of various salts on tryptophan synthesis from d-serine.

**Salts**	**tryptophan synthesis**
NH_4_Cl	–
NH_4_NO_3_	–
NH_4_H_2_PO_4_	–
(NH_4_)_2_HPO_4_	+
(NH_4_)_2_CO_3_	–
K_2_HPO_4_	–
Na_2_HPO_4_	–
NaCl	–
Seawater	–

Note: – no synthesis, + synthesis.
